# High-Temperature X-Ray Crystal Structure Analysis of Schiff Base Cu(II) and Ni(II) Complexes and Data Statistics

**DOI:** 10.3390/molecules30061289

**Published:** 2025-03-13

**Authors:** Anna Okui, Rin Tsuchiya, Daisuke Nakane, Takashiro Akitsu, Toby J. Blundell

**Affiliations:** 1 Department of Chemistry, Faculty of Science, Tokyo University of Science, 1-3 Kagurazaka, Shinjuku-ku, Tokyo 162-8601, Japana29776@rs.tus.ac.jp (D.N.); 2 Department of Chemistry, Durham University, South Road, Durham DH1 3LE, UK; toby.j.blundell@durham.ac.uk

**Keywords:** Schiff base, nickel, copper, high temperature, displacement parameters

## Abstract

In this study, single crystals of previously reported Schiff base copper (II) (**Cu**) and nickel (II) (**Ni**) complexes were synthesized; a structural analysis was performed using data measured at high temperatures, 298 K and 410 K; and CIF and electron density maps were obtained. The purpose of this study was to examine the accuracy of high-temperature measurements in X-ray crystal structure analyses and the details of atomic movement. Various data (statistics such as standard deviation) obtained from the structural analysis, such as the lattice constants, temperature factors, and electron density in cases without phase transitions, were compared. In addition, the anisotropic temperature factors were statistically processed. In the electron density map, the electron density tended to decrease at high temperatures. Looking at the two-dimensional fingerprint plot constructed from the Hirshfeld surface analysis, the intermolecular interactions between chlorine atoms and hydrogen atoms in the **Cu** changed significantly with the temperature change. In addition, the change in the anisotropic temperature factor of chlorine was significant. Moreover, a difference was observed in the analytical data at room temperature and high temperatures, which is thought to be useful for creating a model of temperature dependence.

## 1. Introduction

Few studies have used high-temperature measurements in structural analysis. Such measurements are usually performed at low temperatures to improve data accuracy, and high-temperature measurements are only performed when a phase transition is involved. Regarding such high-temperature measurements, some detailed investigations have been conducted using electron diffraction. Electron diffraction is usually performed at an extremely low temperature of −180 °C, but in [1], a detailed investigation was conducted at 220 °C, and in [2], high-temperature conditions were provided to obtain structural information from anisotropic displacement parameters (ADP) using three-dimensional (3D) electron diffraction. The use of electron diffraction has the following advantages: the electron beam causes less damage to the crystal; a complete 3D single crystal diffraction pattern, rather than a projection, can be obtained, so that the obtained amount of information is greater than that of X-ray powder diffraction; the intensity of the diffracted beam can be high, and although multiple scattering causes interference, it can be improved via dynamic improvements; and hydrogen atoms can be detected. Therefore, the research on high-temperature measurements is progressing [1]. Furthermore, X-ray crystal structure analyses are important because they can obtain the electron density distribution in a substance. However, few investigations have been conducted on the temperature dependence of X-ray crystal structure analyses.

Structural data such as thermal expansion, electron density distribution, and temperature factors are important when examining temperature dependence in detail. Two types of temperature factors exist: isotropic and anisotropic. Isotropic temperature factors (*U*_eq_) have a limited ability to detect possible structural disorders and atomic motion. In contrast, anisotropic temperature factors (ADP and *U*_ij_) provide information on the average displacement of atoms from their typical positions in the crystal, which can provide insight into disorders in and the flexibility of atomic displacements. Thermal vibrations should be assumed to be anisotropic, and *U*_ij_ is modeled by components such as *U*_11_, *U*_12_, *U*_13_, *U*_22_, *U*_23_, and *U*_33_. In the analysis of anisotropic temperature factors [2], certain features should be noted: Some of the axes are negative or zero, with no physical meaning. If no displacements are seen, the atoms should be represented as spheres rather than ellipses. Additionally, strong anisotropic behaviors may be due to a large disorder, the improper processing of data, or experimental errors. Moreover, atoms being stretched out in one direction suggests that the atoms have a discrete structure or are moving more strongly in that direction than in others [3].

Examples of past research into electron density and temperature factors include the following. Upon examining whether a certain experimental method was sufficient for the study of the thermal behavior of molecules, precise absolute measurements using counter methods were confirmed to be preferable over photographic methods when discussing the thermal expansion state [4], confirmed by comparing the standard deviation of the coordinates with the standard deviation of the temperature factors, which showed that the temperature factors depend on higher-order reflections than the coordinates. In addition, the effect of temperature factors is also important for electron density distribution analysis, and it is necessary to accurately consider the effects of thermal vibration by separating the distribution of valence electrons from the displacement due to atomic thermal vibration [5]. Therefore, a highly accurate analysis of temperature factors is necessary. Here, the multipole expansion method makes it easy to analyze the contribution of valence electrons, since it can separate the effect of temperature factors. Regarding the formulation of the temperature factor, the G-C expansion based on statistical theory is adopted in the multipole expansion method [6]. The simplest way to determine the vibrational motion of atoms in a solid is to treat the atoms as harmonic oscillators [7]. However, when the influence of anharmonic thermal vibrations is strong, in cases where the atoms do not have centrosymmetrical structures, the thermal vibrations show non-centrosymmetric anisotropy. This became clear when the accuracy of neutron diffraction improved and temperature factors were precisely analyzed. Since then, anharmonic potentials have been calculated for many substances, and their relationship with phase transitions has been studied by organizing analytical examples [8]. Regarding the influence of thermal vibrations on the X-ray diffraction of perfect single crystals, multiplying the structure factor by the Debye–Waller factor $e^{-M}$ in the dynamical theory seems appropriate when dealing with temperature effects [9].

In addition, the nature of the intermolecular forces is also important. CrystalExplorer’s Hirshfeld surface analysis is a tool that deciphers the intermolecular forces from only the data contained in the crystal structure. This tool is a useful visualization tool that takes into account the deformation of the molecular space in the crystalline environment [10].

Indicators for evaluating the decrease in accuracy in high-temperature measurements include high resolution, high *I*/σ(*I*) values, low *R* values, and high multiplicity. In particular, mapping the density distribution of valence electrons in the innermost reflection and the outermost shell is important. In terms of the maximum diffraction angle *θ*_max_ (or 2*θ*_max_), sin*θ*_max_/*λ* should exceed 0.6 Å⁻^1^. For Mo Kα, a *θ*_max_ of 25° or more is recommended, and for Cu Kα, 67° or more is preferable. It should be noted that if the number of reflection points is small, the number of data available for refinement are limited, resulting in a decrease in accuracy [11].

In this study, we prepared crystal data for two Schiff bases measured at room temperature and high temperature in order to investigate the temperature dependence of crystals in detail based on their X-ray structure data. We then organized and interpreted important data obtained from the structural analysis such as the lattice constants, temperature factors, and electron density (thermal expansion, anisotropic temperature factors, resolution, reflection number, *R* value, Hirshfeld, etc.) in detail. During this process, we also statistically processed the anisotropic temperature factors. The test samples used were the known Cu(C_15_H_12_Cl_2_NO)_2_ and Ni(C_15_H_12_Cl_2_NO)_2_. In addition, to focus only on temperature in this study, the measurements were performed at a high temperature to ensure a lack of phase transitions.

## 2. Materials and Methods

### 2.1. X-Ray Crystallography

**Cu** and **Ni** crystals were obtained in a similar manner to that described in [12,13]. High-temperature single-crystal X-ray crystallography was performed on equipment at Durham University. The equipment used to collect the X-ray crystallography data was a Bruker D8Venture (Billerica, MA, USA) equipped with a focusing mirror Photon III MM C7 CPAD detector, an IμS-III-microsource using MoKα radiation (λ = 0.71073 Å), and a Cryostream Cryosystems 700+ (Oxford, Oxford, UK) open-flow nitrogen cryostat. The structure was solved using Olex2 (https://www.olexsys.org/olex2/, accessed on 10 March 2025) [14] with the ShelXT (http://www.shelx.org/, accessed on 10 March 2025) [15] structure solution program using Intrinsic Phasing and refined with the ShelXL [15] refinement package using least-squares minimization of F^2^. Non-hydrogen atoms were refined with anisotropic displacement parameters, and hydrogen atoms were placed on different maps and modeled isotropically with a riding model unless otherwise specified. The crystallographic data for the structure have been deposited at the Cambridge Crystallographic Data Centre (12 Union Road Cambridge CB2 1EZ UK; https://www.ccdc.cam.ac.uk/, accessed on 10 March 2025) with deposition numbers CCDC-2416914-2416917.

The crystallographic data for **Cu** 298 K, **Cu** 410 K, **Ni** 298 K, and **Ni** 456 K are summarized in Table 1. No significant changes due to temperature were found in the structures of either **Cu** or **Ni**. As investigated in [12,13], analogous metal complexes often show phase transitions and change crystal coordination geometries at high temperatures, which is basically the reason for serious thermal displacement.

### 2.2. Calculations

The crystal structures of **Cu** and **Ni** were calculated using the Gaussian 09W software package Revision D.02 (Gaussian, Inc., Wallingford, CT, USA) [16] with a Windows 11 personal computer. Frequency calculation (Freq) was selected as the type of calculation. Density functional theory (DFT) was used together with 3 of the 11 functionals in B3LYP for all calculations because of the balance between the calculation accuracy and time. The basis set 6-311G (d) was applied to all atoms. “Int=grid=ultrafine” was used as an additional keyword to improve the accuracy of numerical integration in the DFT calculations.

GaussView5 was used for analysis and visualization of the calculation results. All calculations were carried out under gas phase (isolated) conditions.

The CrystalExplorer 17.5 (https://crystalexplorer.net/, accessed on 10 March 2025) [10] program was used for the Hirshfeld surface analyses and fingerprint plots [17,18]. The Hirshfeld surface was represented by the normalized contact distance (*d_norm_*). If this distance is shorter than the van der Waals radius, it is shown in red, and if it is longer, it is shown in blue. In the two-dimensional (2D) fingerprint plots, de was plotted on the vertical axis and di was plotted on the horizontal axis.

## 3. Results

### 3.1. Brief Description of Crystal Structures

**Figure 1:**
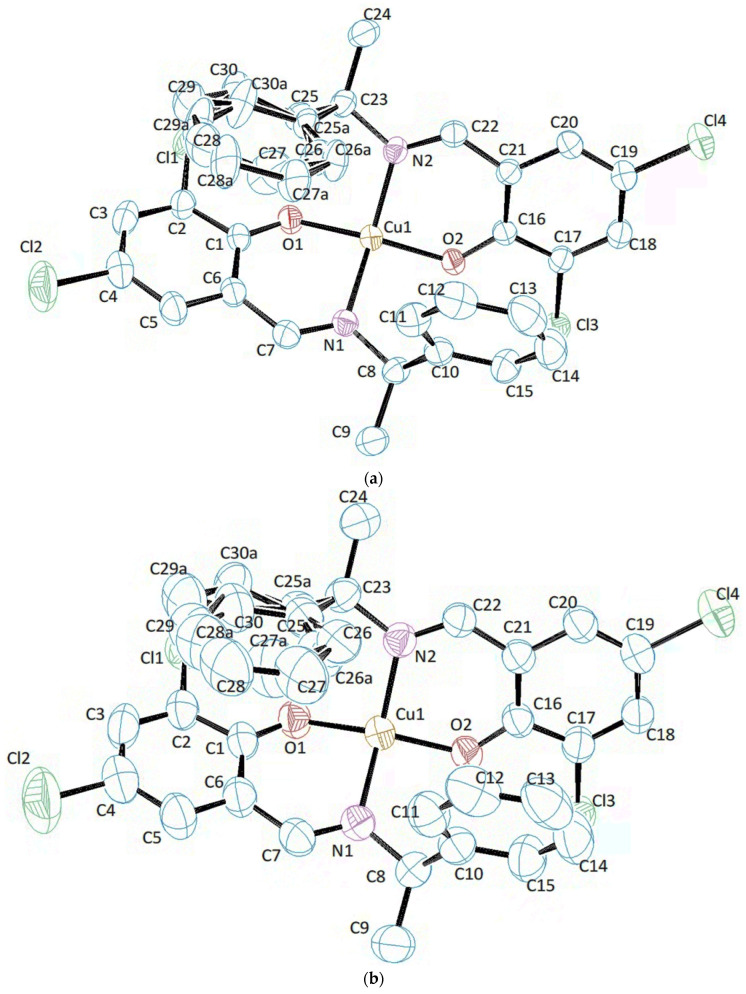

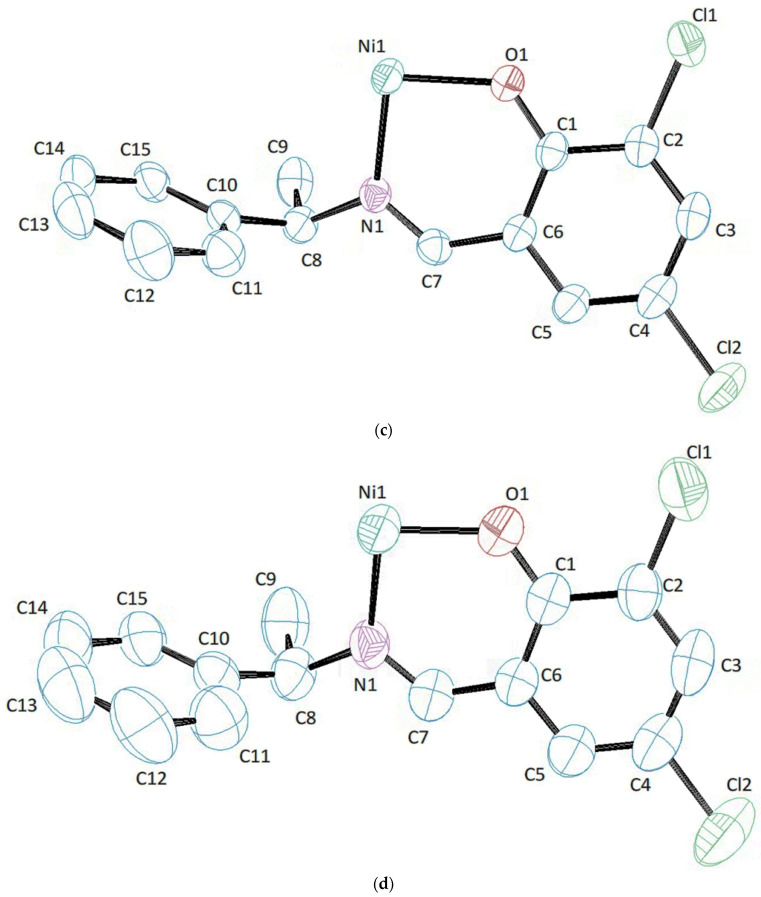
The molecular structure and the asymmetric unit show the atom-labelling scheme: (**a**) **Cu** 298 K; (**b**) **Cu** 410 K; (**c**) **Ni** 298 K; (**d**) **Ni** 456 K. Displacement ellipsoids are drawn at the 50% probability level. H atoms are omitted for clarity.

The molecular structures of **Cu** 298 K and **Cu** 410 K are depicted in Figure 1, and their selected bond distances and angles are given in Table 2. The phenyl group of the **Cu** was disordered, which was treated with SHELXL on Olex2 in a normal way. For example, for **Cu** 298 K, positional disorder was observed for the C25-C30 phenyl group and refined to an occupancy of 0.71(6):0.29(6) for parts 1 and 2. Relevant 1,2- and 1,3-distances were restrained to be approximately equal. Enhanced rigid bond restraints (RIGUs) were applied to the disordered components.

As can be seen from Table 1, the structure itself does not change due to temperature, so we looked at the bond lengths and angles (Table 2 and Table 3). Additionally, no significant change was seen in the torsion angle, so please refer to the deposited CCDC data for detailed results.

**Figure 2:**
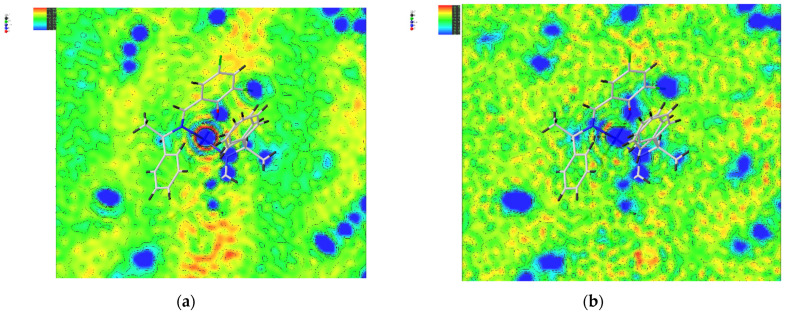

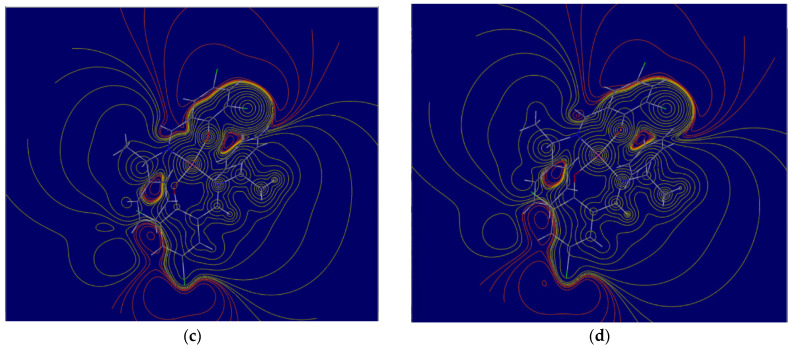
The electron density map of **Cu** 298 K and **Cu** 410 K, using Gaussian09 and the measured values: (**a**) map of the measured value of **Cu** 298 K; (**b**) map of the measured value of **Cu** 410 K; (**c**) Gaussian09 map of **Cu** 298 K; (**d**) Gaussian09 map of **Cu** 410 K.

**Figure 3:**
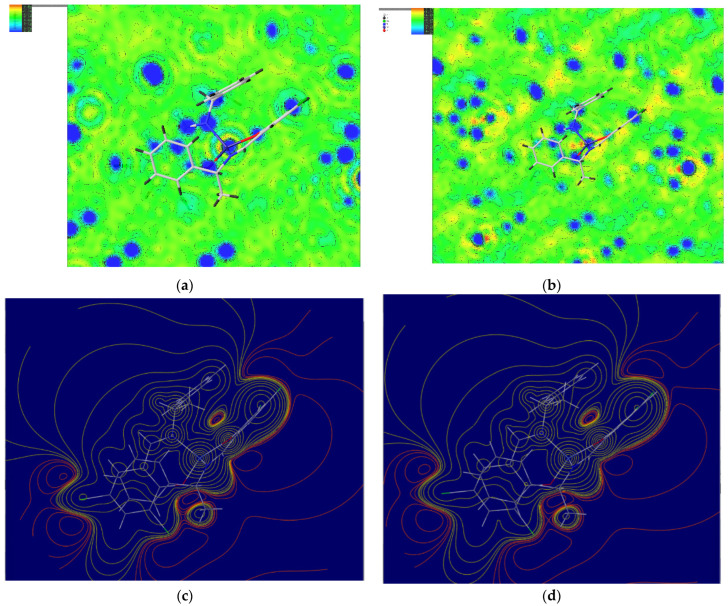
The electron density map of **Ni** 298 K and **Ni** 456 K, using Gaussian09 and the measured values: (**a**) map of the measured value of **Ni** 298 K; (**b**) map of the measured value of **Ni** 456 K; (**c**) Gaussian09 map of **Ni** 298 K; (**d**) Gaussian09 map of **Ni** 456 K.

The quality of the data was confirmed by focusing on the number of reflections, *R* values, and *S* values used in the measurement. As seen in Table 1, for both **Cu** and **Ni**, the number of effectively available strong reflections decreased at higher temperatures. The *R* and *S* values increased at higher temperatures, showing that the accuracy of the data had decreased. However, the total number of reflections and the number of independent reflections for **Cu** increased. This large number of reflections is because this collection has been refined on more data (to a higher angle). Additionally, the data quality for the high-temperature **Ni** structures appears to be more affected by increasing temperature than **Cu**: for **Ni** 456 K, several high-angle reflections have been omitted from the data, as the reflections were no longer observed (and essentially showed up as 0 in *F*_obs_). For both **Cu** and **Ni**, electron density maps (Figure 2 and Figure 3) were drawn based on the density functional theory (DFT) calculations and the analysis of measurements. The regions predicted to have high electron density based on calculations also had high densities in the actual measurements and analysis. At higher temperatures, the electron density became more widespread and thus lower.

Table 4 shows the rates of change in axis length and volume for both complexes when heated at room temperature. **Cu** tends to expand along the *b*- and *c*-axes, while **Ni** tends to expand along the *c*-axis. The reason for both complexes tending to expand along the *c*-axis is thought to be because there is space available. The fact that **Cu** tends to expand along the *b*-axis is thought to be related to the presence of a disorder in **Cu**.

### 3.2. Intermolecular Interactions

**Figure 4:**
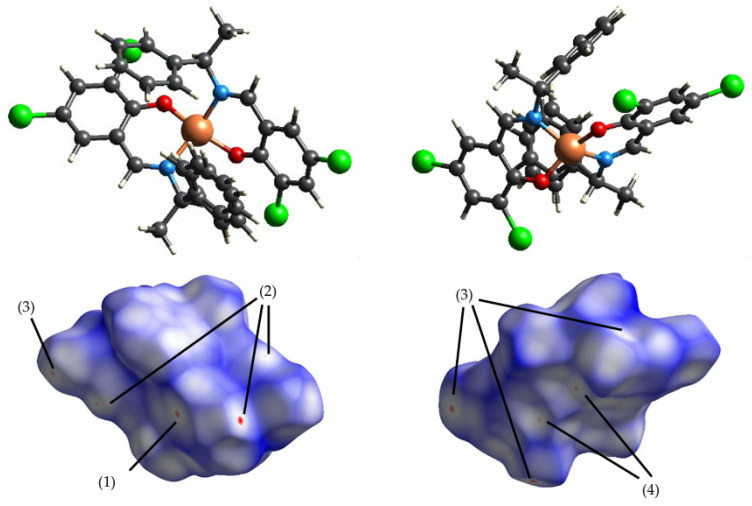
Hirshfeld surface of **Cu** 298 K mapped by *d_norm_*.

**Figure 5:**
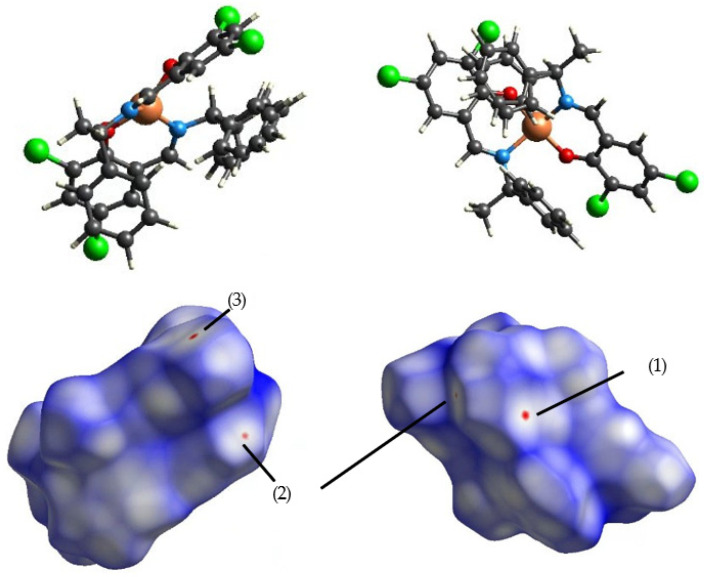
Hirshfeld surface of **Cu** 410 K mapped by *d_norm_*.

**Figure 6:**
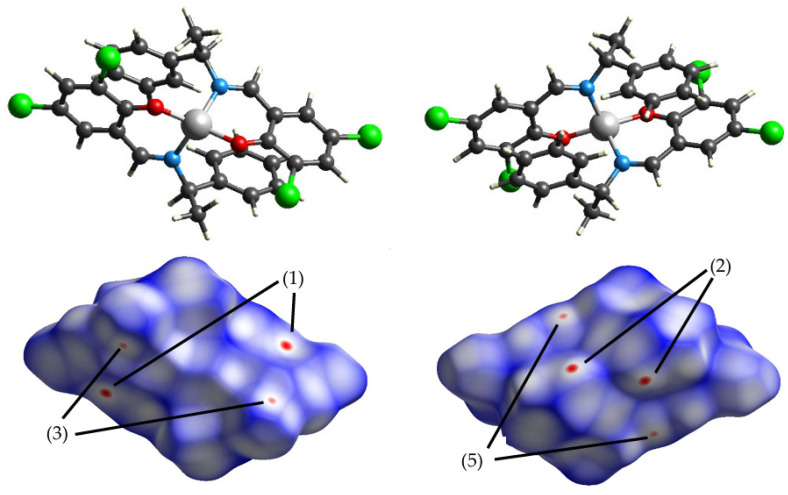
Hirshfeld surface of **Ni** 298 K mapped by *d_norm_*.

**Figure 7:**
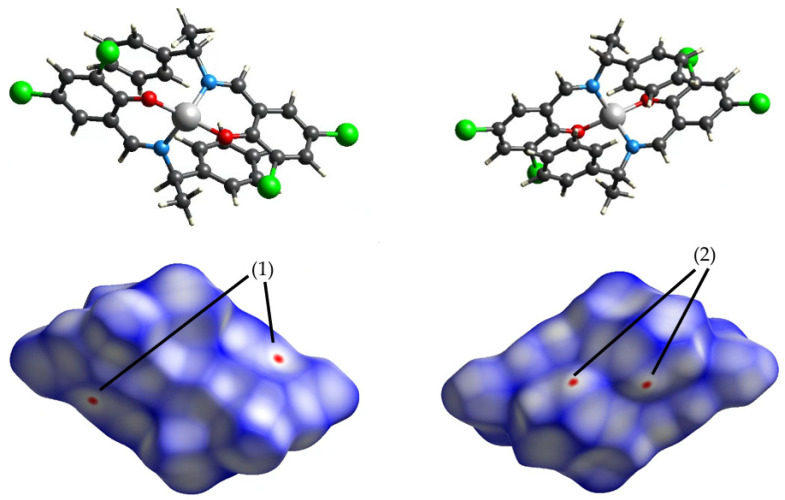
Hirshfeld surface of **Ni** 456 K mapped by *d_norm_*.

The calculated Hirshfeld surfaces are shown in Figure 4, Figure 5, Figure 6 and Figure 7. The points that contributed to the surface in both **Cu** and **Ni** can be seen in the red regions (shorter than the van der Waals radius).

The number (1) represents the interaction between π-conjugated systems; (2) represents the CH-π interaction between aromatic rings and H atoms; (3) represents the interaction between chlorine, with ample space, and the H atom; (4) represents the hydrogen bond between oxygens; and (5) represents the carbon side.

**Figure 8:**
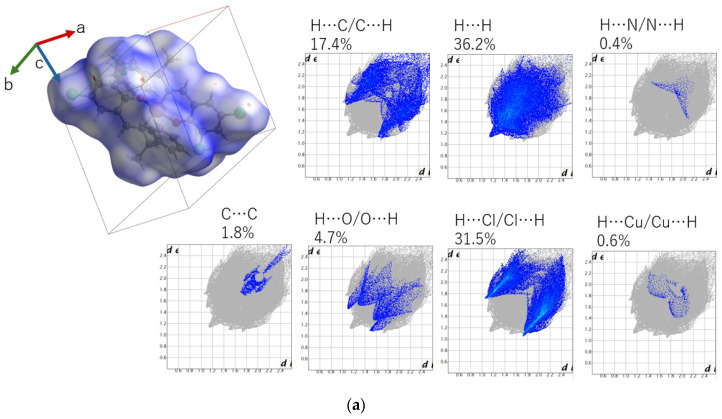

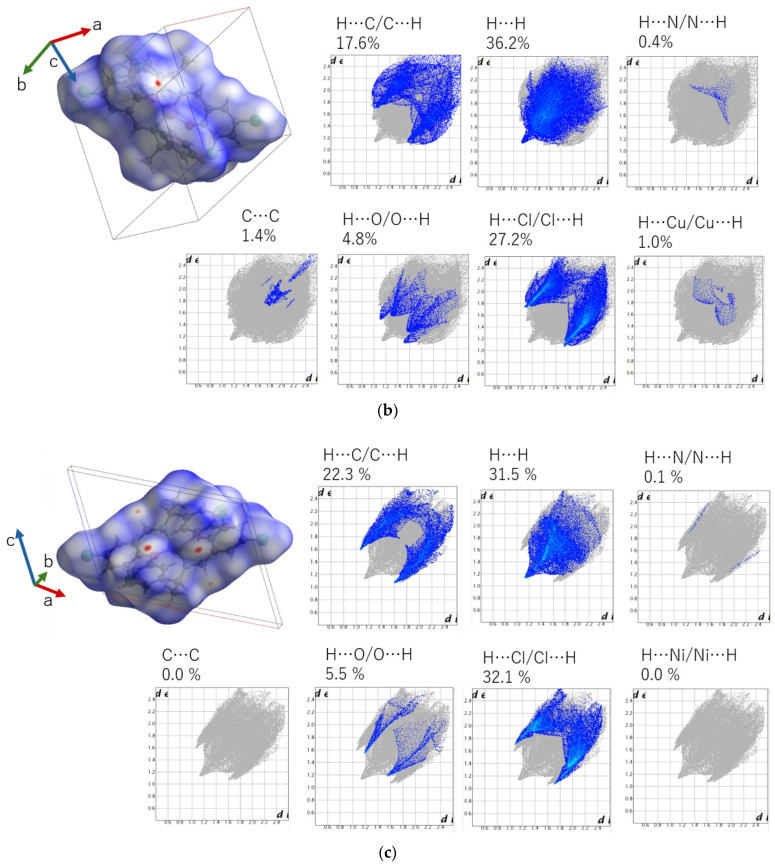

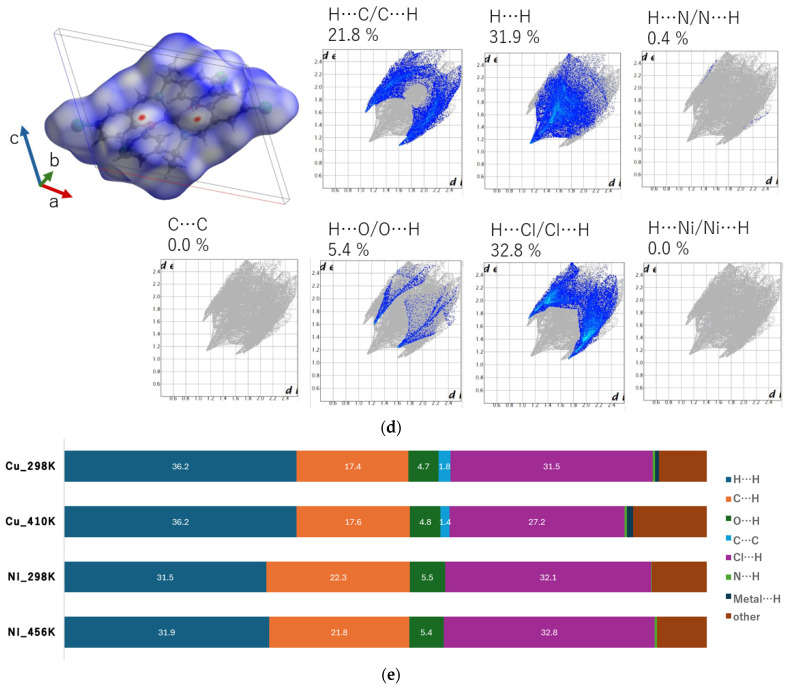
Two-dimensional fingerprints (horizontal axis *d*i and vertical axis *d*ε) (**a**) **Cu** 298 K; (**b**) **Cu** 410 K; (**c**) **Ni** 298 K; and (**d**) **Ni** 456 K, and (**e**) the percentage contribution of the interaction plotted as a bar graph.

Figure 8 shows the contribution of each molecule to intermolecular interactions in a bar graph. The intermolecular interactions of chlorine and hydrogen in **Cu** are more affected by temperature than those of **Ni**.

## 4. Discussion

### 4.1. Sakurai Comparison

Next, we compared the anisotropic temperature factors. However, the standard deviation increases with increasing temperature over time. Therefore, numerical comparisons should be conducted with caution. The following Formula (1) of *t* value, used by Sakurai [19], allows us to determine whether the difference is significant when comparing measurements with standard deviations:

(1) $$t=\frac{\left|d_{1}-d_{2}\right|}{{\left\{\sigma^{2}\left(d_{1}\right)-\sigma^{2}\left(d_{2}\right)\right\}}^{\frac{1}{2}}}$$

where $d_{1}$ and $d_{2}$ are measurements with the standard deviations $\sigma \left(d_{1}\right)$ and $\sigma \left(d_{2}\right)$.

**Theorem** **1.** *A formula devised by Sakurai to determine t value whether a difference is significant when comparing a measured value with the standard deviation.*

A *t* value less than 2 is meaningless, a value between 2 and 2.5 is probably meaningful, and a value greater than 2.5 is meaningful.

### 4.2. Statistical Discussion of Temperature Factors

The comparison of anisotropic temperature factors at room temperature and high temperature is shown in Table 5, Table 6 and Table 7 below, in which bold values should be noted. In the column with the difference in displacement, displacement values in the top 10% of values for all atoms in each complex are underlined. In the column with the *t* value, values of 2 or greater are underlined, and those 2.5 or greater are also bolded.

First, for the central metal, as shown in Table 5, significant differences were found in the *a*-, *b*-, and *c*-axes in both **Cu** and **Ni**. For the carbon of the methyl group, as shown in Table 6, significant differences were found in the *a*-, *b*-, and *c*-axes for **Cu**, but only in the b-axis direction for **Ni**. For chlorine, as shown in Table 7, the difference in displacement in both **Cu** and **Ni** was large, particularly Cl2, because chlorine was in a spatially empty place. In addition, both the large displacements in the *b*- and *c*-axes match the direction of thermal expansion. This may also be related to the large change in the intermolecular interaction of chlorine and hydrogen in **Cu** in the two-dimensional fingerprint. However, since no structural changes were seen, such as in the bond angles, the significant differences are merely a statistical variation.

The phenyl group of **Cu** was disordered; this is thought to be due to the difference in crystal packing, which gives the phenyl group more space to rotate or “wobble” compared with **Ni**. This disorder causes a symmetry break from **Ni** (*Z*’ = 0.5) to **Cu** (*Z*’ = 1). **Ni** also shows some phenyl group wiggle, but not as much as in **Cu**, so there is no disorder. Therefore, the anisotropic temperature factors for **Cu** are not discussed in detail in this article.

## 5. Conclusions

The known copper (II) complex (**Cu**) was measured at 298 K and 410 K, and the nickel (II) complex (**Ni**) was measured at 298 K and 456 K. **Cu** tended to expand in the direction of the b- and c-axes, while **Ni** tended to expand in the direction of the c-axis. No phase transition was involved. Even though the resolution was almost the same, at high temperatures, **Cu** showed a high number of reflections, while **Ni** showed a decrease in the number of independent reflections. The analysis at high and low temperatures showed no noticeable difference in bond distances or bond angles. The electrons became less dense overall at high temperatures. In the Hirshfeld analysis (hydrogen was represented using a riding model), a significant difference in Cl-H interactions due to temperature was observed for **Cu**, but not for **Ni**. When the difference in anisotropic temperature factors was evaluated, a significant difference was observed for chlorine due to temperature, which was the cause of the difference in Cl-H interactions. The phenyl group of **Cu** was disordered, which was due to the environment around the phenyl group. In this way, high-temperature measurements and various data statistics revealed that for atoms to obtain significant thermal vibrations, specific conditions are required.

The data precision decreased more significantly for **Ni** than for **Cu**. As such, even with the same structure, the degree of precision decrease may vary depending on the central metal, so care must be taken.

This detailed investigation of the temperature dependence of X-ray crystal structure analysis is likely to be useful in creating models of temperature dependence. In fact, in [20], we used X-ray and neutron diffraction data to create a model that divides ADP measured as a function of temperature into its temperature-dependent and temperature-independent contributions. In this study, data other than ADP were also analyzed in detail, which is likely to contribute to the creation of models that reflect more factors.

In electron diffraction and quantum crystallography, high-precision approximations of the electron density are of interest, but much more information remains to be gained from the (intentionally high) temperature factors of X-ray diffraction and statistical considerations of the data. In the future, we will conduct a deeper study by visualizing the anisotropic temperature factors that have been statistically processed.

## Figures and Tables

**Table 1:** Crystallographic data for **Cu** 298 K, **Cu** 410 K, **Ni** 298 K, and **Ni** 456 K.

	Cu 298 K	Cu 410 K	Ni 298 K	Ni 456 K
Formula	Cu(C_15_H_12_Cl_2_NO)_2_	Cu(C_15_H_12_Cl_2_NO)_2_	Ni(C_15_H_12_Cl_2_NO)_2_	Ni(C_15_H_12_Cl_2_NO)_2_
Crystal system	Monoclinic	Monoclinic	Monoclinic	Monoclinic
Space group	*P*2_1_	*P*2_1_	*C*2	*C*2
*Z*	2	2	2	2
*a* (Å)	8.6444(2)	8.6632(6)	13.9514(7)	14.043(14)
*b* (Å)	14.8662(4)	14.9097(12)	10.9409(6)	11.049(11)
*c* (Å)	11.1747(3)	11.2065(9)	10.1046(5)	10.261(10)
*β* (°)	97.8490(10)	98.267(7)	117.4460(10)	118.24(3)
*V* (Å)	1422.60(6)	1432.45(19)	1368.77(12)	1402(2)
ρ_calc_ (g/cm^3^)	1.517	1.507	1.565	1.527
μ (mm^−1^)	1.175	1.167	1.132	1.105
*F*(000)	662.0	662	660	660
Total reflections	33,450	48,402	20,679	5194
2*θ* at total reflection	25.242	25.242	25.242	25.242
Independent reflection	7994	13,084	3815	3150
Strong reflection that satisfies *I* > 2σ(*I*)	6565	6551	3094	2041
*R*_obs_	0.0382	0.0734	0.0323	0.0626
*wR*_obs_	0.0723	0.1592	0.0534	0.15
*R*_all_	0.0538	0.1532	0.0518	0.0986
*wR*_all_	0.0802	0.199	0.0594	0.1753
S	1.032	1.06	1.043	1.066
⟨*I*/σ(*I*)⟩	0.0403	0.0462	0.0475	0.0733

**Table 2:** Selected geometric parameters (Å, °) for **Cu** 298 K and **Cu** 410 K.

	**Cu** **298 K**	**Cu** **410 K**
Cu1–O1	1.893(3)	1.893(3)
Cu1–O2	1.882(3)	1.882(3)
Cu1–N1	2.001(3)	2.001(3)
Cu1–N2	1.983(3)	1.983(3)
O1–C1	1.298(4)	1.288(8)
O2–C16	1.295(4)	1.279(7)
N1–C7	1.287(5)	1.289(9)
N2–C22	1.292(5)	1.282(8)
N1–C8	1.477(5)	1.475(9)
N2–C23	1.487(5)	1.475(8)
C6–C7	1.438(6)	1.422(11)
C22–C21	1.439(5)	1.430(9)
C8–C9	1.532(6)	1.517(11)
C23–C24	1.526(6)	1.515(10)
C8–C10	1.521(6)	1.527(10)
C23–C25	1.516(9)	1.52(2)
C23–C25A	1.528(19)	1.504(12)
	**Cu** **298 K**	**Cu** **410 K**
O1–Cu1–O2	150.7(2)	150.88(12)
O1–Cu1–N1	92.00(13)	92.1(2)
O2–Cu1–N1	94.57(13)	94.8(2)
O1–Cu1–N2	97.23(12)	97.3(2)
O2–Cu1–N2	93.73(12)	93.8(2)
Cu1–O1–C1	124.3(2)	124.3(4)
Cu1–O2–C16	127.9(2)	128.0(4)
Cu1–N1–C7	120.9(3)	120.0(5)
Cu1–N2–C22	122.5(3)	121.9(4)
Cu1–N1–C8	117.7(3)	117.9(4)
Cu1–N2–C23	117.2(2)	117.3(4)
N1–C7–C6	126.7(4)	127.3(7)
N2–C22–C21	126.6(4)	127.0(6)
C7–N1–C8	120.7(4)	121.4(6)
C22–N2–C23	120.0(3)	120.5(6)
N1–C8–C9	114.7(3)	115.0(6)
N2–C23–C24	115.4(3)	115.2(6)
C9–C8–C10	111.9(3)	111.8(6)
C24–C23–C25	110.7(17)	115(6)
C24–C23–C25A	114(4)	110.3(17)
O1–C1–C6	123.8(4)	123.9(6)
O2–C16–C21	124.4(3)	124.1(6)
O1–C1–C2	120.4(4)	120.6(7)
O2–C16–C17	119.1(3)	119.2(6)

**Table 3:** Selected geometric parameters (Å, °) for **Ni** 298 K and **Ni** 410 K.

	**Ni** **298 K**	**Ni** **456 K**
Ni1–O1	1.9120(19)	1.898(6)
Ni1–N1	2.008(2)	1.994(7)
Cl1–C2	1.746(3)	1.732(10)
Cl2–C4	1.744(3)	1.744(10)
O1–C1	1.298(3)	1.301(10)
N1–C7	1.286(3)	1.288(10)
N1–C8	1.498(4)	1.505(10)
C6–C7	1.440(4)	1.414(11)
C8–C9	1.517(5)	1.537(15)
C8–C10	1.521(6)	1.508(13)
	**Ni** **298 K**	**Ni** **456 K**
O1–Ni1–O1	141.50(13)	140.5(4)
O1–Ni1–N1	107.45(9)	107.6(3)
O1–Ni1–N1	92.73(9)	92.9(3)
Ni1–O1–C1	125.87(19)	126.0(6)
Ni1–N1–C7	121.09(19)	121.2(6)
Ni1–N1–C8	123.01(18)	123.7(5)
N1–C7–C6	127.9(3)	128.6(9)
C7–N1–C8	115.9(2)	115.2(7)
N1–C8–C9	108.6(3)	107.3(8)
N1–C8–C10	110.5(2)	111.1(7)
C9–C8–C10	115.2(3)	115.3(8)
O1–C1–C6	124.3(2)	124.0(7)
O1–C1–C2	120.1(3)	119.8(9)

**Table 4:** Ratios of axis length and volume for **Cu** (410 K/298 K) and **Ni** (456 K/298 K).

	Cu	Ni
*a*-axis	1.0022	1.0066
*b*-axis	1.0029	1.0099
*c*-axis	1.0028	1.0155
volume *V*	1.0069	1.0243

**Table 5:** Anisotropic temperature factor of the central metal.

Label	Cu1				Ni1			
	Cu 298 K	Cu 410 K	Difference	*t*	Cu 298 K	Cu 410 K	Difference	*t*
*U*_11_	27.6(2)	41.9(3)	14.3	**3.97**	25.4(3)	44.1(7)	18.7	2.46
*U*_22_	35.7(2)	58.3(4)	22.6	**5.05**	41.8(3)	72.8(9)	31.0	**3.27**
*U*_33_	31.1(2)	51.9(4)	20.8	**4.65**	31.3(3)	53.4(8)	22.1	**2.59**
*U*_23_	−1.5(2)	−2.0(4)	−0.5	0.11	0	0		
*U*_13_	0.50(16)	2.0(2)	1.5	0.09	12.7(2)	21.6(6)	8.9	1.41
*U*_12_	2.5(2)	2.8(4)	0.3	0.07	0	0		

**Table 6:** Anisotropic temperature factor of the methyl group carbon.

**Label**	**C9**				**C9**			
	**Cu** **298 K**	**Cu** **410 K**	**Difference**	***t***	**Ni** **298 K**	**Ni** **456 K**	**Difference**	***t***
*U*_11_	45(2)	67(5)	22.0	**4.09**	45.8(18)	76(6)	30.2	1.59
*U*_22_	54(3)	81(6)	27.0	**4.02**	71(3)	129(11)	58.0	**5.09**
*U*_33_	45(3)	83(5)	38.0	**6.52**	32.0(16)	58(6)	26.0	1.52
*U*_23_	−12(2)	−20(4)	−8.0	1.79	6.8(17)	18(7)	11.2	0.61
*U*_13_	8(2)	16(4)	8.0	1.79	13.0(14)	17(5)	4.0	0.27
*U*_12_	−1(2)	2(4)	3.0	0.67	−11.9(18)	−20(7)	−8.1	0.42
**Label**	**C24**							
	**Cu** **298 K**	**Cu** **410 K**	**Difference**	***t***				
*U*_11_	55(3)	77(5)	22.0	**3.77**				
*U*_22_	46(3)	80(5)	34.0	**5.83**				
*U*_33_	34(2)	59(4)	25.0	**5.59**				
*U*_23_	−4.4(19)	−5(4)	−0.6	0.03				
*U*_13_	6(2)	12(4)	6.0	1.34				
*U*_12_	6(2)	10(4)	4.0	0.89				

**Table 7:** Anisotropic temperature factor of chlorine.

**Label**	**Cl1**				**Cl1**			
	**Cu** **298 K**	**Cu** **410 K**	**Difference**	***t***	**Ni** **298 K**	**Ni** **456 K**	**Difference**	***t***
*U*_11_	55.7(7)	81.7(14)	26.0	1.66	43.7(5)	72.3(16)	28.6	1.71
*U*_22_	51.7(7)	83.6(15)	31.9	1.93	57.7(6)	105(2)	47.3	**7.48**
*U*_33_	58.4(7)	91.8(15)	33.4	2.02	42.5(5)	74.6(17)	32.1	1.81
*U*_23_	−15.6(6)	−22.3(12)	−6.7	0.50	−12.5(4)	−21.5(15)	−9.0	0.58
*U*_13_	−8.9(6)	−12.2(12)	−3.3	0.25	13.5(4)	20.8(13)	7.3	0.54
*U*_12_	−4.1(6)	−6.3(11)	−2.2	0.18	−8.5(4)	−16.3(15)	−7.8	0.50
**Label**	**Cl2**				**Cl2**			
	**Cu** **298 K**	**Cu** **410 K**	**Difference**	***t***	**Ni** **298 K**	**Ni** **456 K**	**Difference**	***t***
*U*_11_	87.1(11)	126(3)	38.9	**3.41**	36.9(5)	62.8(16)	25.9	1.55
*U*_22_	79.4(11)	125(2)	45.6	**4.08**	91.5(8)	162(3)	70.5	**8.25**
*U*_33_	74.1(10)	118(2)	43.9	**4.30**	64.5(6)	112(2)	47.5	**7.51**
*U*_23_	22.5(8)	33.1(19)	10.6	0.51	10.5(5)	21(2)	10.5	1.95
*U*_13_	−25.9(9)	−37.0(19)	−11.1	0.53	19.1(4)	31.5(16)	12.4	0.75
*U*_12_	22.3(9)	31(2)	8.7	0.94	23.5(5)	40.8(18)	17.3	0.93
**Label**	**Cl3**							
	**Cu** **298 K**	**Cu** **410 K**	**Difference**	***t***				
*U*_11_	44.1(6)	64.6(10)	20.5	1.76				
*U*_22_	88.5(9)	137(2)	48.5	**5.26**				
*U*_33_	29.9(5)	49.4(8)	19.5	2.07				
*U*_23_	1.4(5)	2.6(10)	1.2	0.11				
*U*_13_	8.4(4)	10.3(8)	1.9	0.21				
*U*_12_	3.2(6)	4.2(11)	1.0	0.08				
**Label**	**Cl4**							
	**Cu** **298 K**	**Cu** **410 K**	**Difference**	***t***				
*U*_11_	27.8(5)	42.3(8)	14.5	1.54				
*U*_22_	59.8(7)	95.7(15)	35.9	2.17				
*U*_33_	59.4(7)	97.8(15)	38.4	2.32				
*U*_23_	4.6(6)	7.6(12)	3.0	0.22				
*U*_13_	−0.6(5)	1.6(9)	2.2	0.21				
*U*_12_	1.4(5)	2.2(9)	0.8	0.08				

## Data Availability

No new data were created or analyzed in this study. Data sharing is not applicable to this article.
